# Influence of biological maturation status on selected anthropometric and physical fitness variables in adolescent male volleyball players

**DOI:** 10.7717/peerj.13216

**Published:** 2022-04-05

**Authors:** Mario Albaladejo-Saura, Raquel Vaquero-Cristóbal, Juan A. García-Roca, Francisco Esparza-Ros

**Affiliations:** 1 Kinanthropometry International Chair, Universidad Católica San Antonio, Murcia, Murcia, Spain; 2 Faculty of Sport Science, Universidad Católica San Antonio, Murcia, Murcia, Spain

**Keywords:** Body composition, Development, Growth, Sport performance, Talent identification, Team sport

## Abstract

**Background:**

The identification of sport talent among adolescent athletes is a topic that in recent years has been a major focus of interest for both the scientific community and sport managers. Both anthropometry and physical performance through fitness tests have demonstrated to be key elements. Biological maturation, due to its influence on anthropometric variables and physical fitness, has also been studied in relation to sport talent identification.

**Objective:**

To analyse differences according to biological maturation status in anthropometric characteristics and performance in physical fitness tests, and to determine which variables predict better performance in physical fitness tests in adolescent volleyball players.

**Methods:**

A cross-sectional design was followed to collect the data. A total of 48 male sub-elite volleyball players (14.17 ± 0.73 years) completed a socio-demographic and sports ad hoc questionnaire. Anthropometric variables were measured following the guidelines of the International Society for the Advancement in Kinanthropometry (ISAK) including four basic measurements (body mass, height, sitting height and arm span); eight skinfolds (triceps, biceps, subscapular, iliac crest, supraspinale, abdominal, thigh and calf); four girths (arm relaxed, flexed and tensed arm, middle thigh and calf); five breadths (biacromial, biileocrestal, humerus, femur and bi-styloid); three lengths (acromiale-radiale, radiale-stylion and stylion-medio dactilion); and a height (ilioespinale). Physical fitness was assessed, including the sit-and-reach, back scratch, long jump, medicine ball throw, counter movement jump (CMJ), 20 meters sprint, and agility tests. Furthermore, maturity offset and age at peak height velocity (APHV) was calculated.

**Results:**

Significant differences were found in the body mass (Mean Difference, MD = 20.86–30.75), height (MD = 11.72–19.09), sitting height (MD = 4.27–10.27), arm span (MD = 12.91–20.78), body mass index (MD = 3.72–5.63), upper limb length (MD = 7.76), corrected muscle girths (MD = 2.06–9.31), ∑6 and 8 skinfolds (MD = 3.67–50.21) fat mass and percentage (MD = 0.30–11.58), muscle (MD = 4.13–10.64) and bone mass (MD = 1.61–3.54) (*p* < 0.001–0.030), showing higher values the early maturers. In the physical fitness tests, significant differences were observed in the medicine ball throw (MD = 1.26–2.80) and in CMJ power (MD = 156.71–379.85) (*p* < 0.001). Regression models identified fat mass percentage predicted worse physical test performance (*p* < 0.001), while age, maturation offset, muscle and bone variables were predictors of better physical performance (*p* < 0.001).

**Conclusions:**

Significant differences based upon the stages of biological maturation were found in the anthropometric and physical condition variables in favor of the players whose maturation process was more advanced, with the variables related to fat and adipose, muscle and bone development conditioning their performance in the physical condition tests.

## Introduction

The identification of sport talent among adolescent athletes is a topic that in recent years has been a major focus of interest for both the scientific community and sport managers (Hertzog et al., 2018). This interest is due to the fact that the implementation of early talent identification programmes can bring advantages to the clubs that carry them out, both in economic and sporting terms regarding the incorporation of young players into top-level teams or long-term economic security (Pion et al., 2015).

Both anthropometric variables, understood as the application of measurements to the study of body size, shape, proportion, composition, maturation and function, with the purpose of aiding to understand the human movement in the context of growth, exercise, performance and nutrition (Ross et al., 1980), and the analysis of physical performance through fitness tests have been key elements in sports talent identification programmes, as previous research has observed the influence they have on elite performance in different sport disciplines (Arede et al., 2019; López-Plaza et al., 2017b). However, it must be taken into account that changes occur during the maturation stage that can affect both anthropometric and physical fitness variables, so in recent decades researchers have paid close attention to the relationship between biological maturation and these variables (Albaladejo-Saura et al., 2021).

Biological maturation has been described as the time required and the process of change until the adult stage of development is reached (Malina & Bouchard, 1991). Among the methods for monitoring biological maturation, the calculation of the age at peak height velocity (APHV) is one of the most widely used indicators (Malina & Bouchard, 1991; Mirwald et al., 2002), and more specifically, the formulas that allow the calculation of APHV based on anthropometric measurements, being a widely used and validated method that have facilitated the assessment of biological maturation in a rapid and non-invasive way (Mirwald et al., 2002).

Most of the studies that have used the APHV to monitor the maturity status of the young athletes divide the sample in groups, classifying as early maturers those athletes who have a lower estimated APHV than the average of the group; late maturers those who have a higher APHV than the average of the group; and on time those players whose estimated APHV is close to the average APHV of the group, depending on the criteria selected (Albaladejo-Saura et al., 2021).

Previous studies in adolescent boys have indicated that biological maturation seems to have a significant relationship with anthropometric and fitness variables, with early male maturers showing higher values in anthropometric variables such as body mass, height, body mass index (BMI), and fat mass percentage; and fitness tests such as medicine ball throw, handgrip strength, counter movement jump (CMJ), and squat jump, probably as a result of hormonal changes that occur during biological maturation (Albaladejo-Saura et al., 2021). Due to volleyball characteristics and rules of play, having greater agility in changes of direction, speed in sprinting actions and greater jumping power, being taller, having a greater arm span or leg length are differentiating elements of top level players (Zhao et al., 2019). All these aspects could be influenced by biological maturation, being necessary to carry out studies that cover this sport modality in order to know the influence of biological maturation in these aspects with the aim of adequately orienting the programmes for the detection of sporting talent in volleyball (Albaladejo-Saura et al., 2021). However, none of these studies have investigated volleyball players (Albaladejo-Saura et al., 2021).

Therefore, the aim of the present research was to analyse the differences according to biological maturation in anthropometric characteristics and performance in physical fitness tests, and to determine which anthropometric variables could predict better performance in physical fitness tests in adolescent volleyball players.

## Materials & Methods

### Subjects

Sample size calculation was performed with software Rstudio (version 3.15.0, Rstudio Inc., Boston, MA, USA). Significance level was set *a priori* at *α* = 0.05. The standard deviation (SD) was set according to the years from peak height velocity from previous studies (SD = 0.65) (Arede et al., 2019). With an estimated error (d) of 0.184 years from peak height velocity, the sample size needed was 48 subjects. Sample was reached by conducting a non-probabilistic convenience sampling, contacting the responsible Regional Federation, which allowed us to include the best four teams in the league classification. A total of 48 1st Regional Division players (age: 14.17 ± 0.73 years) took part in the study.

Before starting the study, coaches, parents and players were informed of the measurement procedures and signed a written informed consent form. Inclusion criteria were: (a) training volleyball regularly, at least two days per week; (b) participating in federated competition; (c) being between 12 and 15 years old; and (d) having played volleyball at least two consecutive seasons at the time of measurement. Participants were excluded in case of: (a) suffering an injury that prevented them from completing the physical fitness tests; and (b) having missed more than 25% of the training sessions in the last three months (Albaladejo-Saura et al., 2020).

### Design

A cross-sectional design was followed, in accordance with the STROBE guidelines. The San Antonio Catholic University granted Ethical approval to carry out the protocol designed for data collection in accordance with the World Medical Association Code (Code number: CE061921). The Declaration of Helsinki statements were followed during the entire process. The measurements were carried out in the players’ usual training sport hall.

### Methodology

An *ad hoc* questionnaire was used to collect socio-demographic and sports information. Questions about the information needed to know if the participants met the inclusion criteria were also included.

The anthropometric assessment was performed following the guidelines of the International Society for the Advancement in Kinanthropometry (ISAK) (Esparza-Ros, Vaquero-Cristóbal & Marfell-Jones, 2019). Four basic measurements (body mass, height, sitting height and arm span) were measured with a SECA 862 scale (SECA, Alemania), a SECA stadiometer (SECA, Germany) and an arm span meter (Smartmet, Mexico) with an accuracy of 0.1 cm, respectively. Eight skinfolds (triceps, biceps, subscapular, iliac crest, supraspinale, abdominal, thigh and calf) were evaluated with a skinfold caliper (Harpenden, UK) with an accuracy of 0.2 mm accuracy. Four girths (arm relaxed, flexed and tensed arm, middle thigh and calf) were measured with an inextensible tape (Lufkin, USA) with 0.1 cm accuracy. Five breadths (biacromial, biileocrestal, humerus, femur and bi-styloid) were taken with an anthropometer (Realmet, Spain) and a small girth sliding caliper (Holtain, UK) with 0.1 cm accuracy. Three lengths (acromiale-radiale, radiale-stylion and stylion-medio dactilion) and a height (ilioespinale) were evaluated with a segmometer (CESCORF, Brazil) with 0.1 cm.

All the measurements were performed by level 2 and 3 anthropometrist accredited by ISAK. The intra- and inter-evaluator technical error of measurement (TEM) were calculated in a sub-sample (*n* = 20). The intra-evaluator TEM was 0.04% in basic measures, lengths, heights and girths; and 1.05% in skinfolds; and the inter-evaluator TEM was 0.06% in basic measures lengths, heights and girths; and 2.87% in skinfolds.

The following measurements were calculated: BMI, fat mass (Slaughter et al., 1988), muscle mass (Poortmans et al., 2005), bone mass (Matiegka, 1921), somatotype (Carter & Heath, 1990), ∑6 skinfolds (triceps, subescapular, supraespinale, abdominal, thigh and calf), ∑8 skinfolds (∑6 skinfolds + biceps and iliac crest,), cormic index [(sitting height/height)*100], upper limb length [acromiale-radiale length + radiale-stylion length + stylion-mediodactilion length], arm corrected girth [relaxed arm girth − (π*triceps skinfold)], thigh [middle thigh girth − (π*thig skinfold)] and leg [calf girth − (π*calf skinfold)] and muscle-bone index [muscle mass/bone mass].

Maturity offset was calculated according to the procedures of Mirwald et al. (Mirwald et al., 2002), using the sex specific formula. This formula has been used in an adolescent athlete population, showing a high interclass correlation coefficient (ICC = 0.96), as well as a low coefficient of variance percentage (CV% = 0.8) and a low typical error (TE = 0.1) (Towlson et al., 2017). The result was used to calculate the age at peak height velocity (APHV) for each subject using the following formula: APHV = chronological age − maturity offset result. The players were classified in three groups, according to the maturity status based on APHV, the early maturers group was composed of players whose APHV was −0.5 years or less with respect to the mean; the average maturers group, whose APHV was ±0.5 years with respect to the mean; and the late maturers group whose APHV was +0.5 years or more with respect to the mean of the group (Wickel & Eisenmann, 2007).

The physical fitness tests were selected according to previously described protocols and performed in the following order: sit-and-reach, with the Acuflex Tester III (Novel Products, U.S.A); back scratch test, with a millimeter ruler (GIMA, Italy); long jump and medicine ball throw with a tape measure (HaeSt, Germany) of 0.1 cm accuracy; CMJ with a force platform (MuscleLab, Norway); 20 m sprint with MySprint (Apple Inc., USA); (Romero-Franco et al., 2017), and agility test (9-3-6-3-9) with five photocells (Microgate, Italy) (Arede et al., 2019; Castro-Piñeiro et al., 2013; Katić, Grgantov & Jurko, 2006; López-Plaza et al., 2017b; Muyor et al., 2014). Before the warm-up, the subjects performed the flexibility tests (Díaz-Soler et al., 2015). This was followed by a standardised warm-up, consisting of 10 min of continuous running, joint mobility and familiarisation with the physical fitness tests. Two researchers with previous experience in the assessment of physical fitness tests were in charge of the familiarisation and assessment of these tests. The same researcher was always in charge of the same tests to avoid inter-evaluator error. The intraclass correlation coefficient (ICC) was 0.995 (95% confidence interval 0.989−0.997), and the coefficient of variation (CV) was 2.3%. The subjects made two attempts at each test, with a two-minute rest between tests. The mean of the two trials was used as final value for subsequent analysis.

### Statistical analysis

The normal distribution of the sample was assessed with the Kolmogorov–Smirnov test, as well as kurtosis, asymmetry, and homogeneity with the Levene test. A descriptive analysis of the sample was carried out. The differences between the maturation groups in the anthropometric variables and the physical fitness tests were analyzed using an ANOVA test, as well as the main effects and interactions of the covariable age including it in an ANCOVA test. Effect size was calculated with partial eta squared (${\eta }_{\mathrm{p}}^{2}$). Bonferroni’s *post hoc* was used to analyse the pairwise differences between groups. The significance level was set *a priori* at *p* < 0.05. The correlations between maturity offset, age, anthropometric and fitness variables were assessed using Pearson’s correlation test in the complete sample and in the sample divided by age groups. After that, a stepwise multiple linear regression with the variables that had shown significant correlations was performed, to find out which variables could predict performance in the physical tests. All statistical analysis was performed with SPSS software (ver 23, IBM, Endicott, NY, US).

## Results

After calculating the APHV, the sample was divided into early (*n* = 8), average (*n* = 33) and late maturers (*n* = 7). The descriptive statistics (mean ± SD) of each group for all measured variables, as well as the differences between maturity groups, the main effects of the covariate age and the interaction maturity group*age can be observed in Table 1.

**Table 1:** Descriptive data and differences according to maturation group, including age covariable main effects and intersection.

Variable	Group (Mean ± SD)			Model								
	Early (*n* = 8)	Average (*n* = 33)	Late (*n* = 7)	Maturation group			Age			Maturation group*age		
				F	*p*	${\eta }_{p}^{2}$	F	*p*	${\eta }_{p}^{2}$	F	*p*	${\eta }_{p}^{2}$
Maturity offset (years)	1.62 ± 0.87	0.46 ± 1.02	0.07 ± 0.74	5.800	0.006	0.205	423.959	<0.001	0.906	376.339	<0.001	0.895
APHV (years)	12.80 ± 0.18	13.56 ± 0.25	14.50 ± 0.58	57.199	<0.001	0.718	3.049	0.088	0.065	376.339	<0.001	0.895
Body mass (Kg)	81.38 ± 13.84	60.51 ± 8.90	50.62 ± 8.25	20.828	<0.001	0.481	15.118	<0.001	0.256	0.129	0.721	0.003
Height (cm)	1.81 ± 0.03	1.70 ± 8.24	1.62 ± 0.07	12.707	<0.001	0.361	26.289	<0.001	0.374	66.324	<0.001	0.601
Arm span (cm)	185.19 ± 3.26	172.28 ± 8.90	164.41 ± 9.67	12.202	<0.001	0.352	13.861	0.001	0.240	52.620	<0.001	0.545
Sitting height (cm)	93.21 ± 2.61	87.21 ± 4.13	82.94 ± 3.18	14.087	<0.001	0.385	88.401	<0.001	0.668	82.143	<0.001	0.651
Upper limb length (cm)	82.36 ± 2.06	76.87 ± 3.73	74.40 ± 4.72	9.959	<0.001	0.307	11.787	0.001	0.211	57.392	<0.001	0.566
Iliospinale height (cm)	102.88 ± 4.71	94.79 ± 12.85	86.87 ± 18.83	2.853	0.068	0.113	7.867	0.007	0.152	0.752	0.390	0.017
Biacromial breadth (cm)	40.75 ± 2.39	36.75 ± 2.06	35.24 ± 2.19	14.734	<0.001	0.396	30.922	<0.001	0.413	25.599	<0.001	0.368
Biiliocrestal breadth (cm)	29.10 ± 1.65	25.69 ± 1.59	24.57 ± 1.57	18.308	<0.001	0.449	16.000	<0.001	0.267	24.211	<0.001	0.355
Femur breadth (cm)	10.54 ± 0.41	9.84 ± 0.43	9.27 ± 0.42	16.725	<0.001	0.426	1.661	0.204	0.036	91.052	<0.001	0.674
Humerus breadth (cm)	7.26 ± 0.44	6.83 ± 0.33	6.55 ± 0.34	7.816	0.001	0.258	10.258	0.003	0.189	45.502	<0.001	0.508
Bi-styloid breadth (cm)	5.51 ± 0.39	5.35 ± 0.28	4.96 ± 0.35	6.354	0.004	0.220	4.469	0.040	0.092	37.628	<0.001	0.461
Corrected arm girth (cm)	26.38 ± 2.72	22.72 ± 2.35	20.44 ± 1.65	12.759	<0.001	0.362	6.970	0.011	0.137	5.248	0.027	0.107
Corrected thigh girth (cm)	49.07 ± 5.08	44.35 ± 3.49	39.76 ± 2.76	11.843	<0.001	0.345	11.403	0.002	0.206	8.044	0.007	0.155
Corrected leg girth (cm)	34.22 ± 1.32	31.63 ± 2.08	29.57 ± 1.57	11.318	<0.001	0.335	20.558	<0.001	0.318	22.974	<0.001	0.343
Endomorphy	3.74 ± 2.01	2.57 ± 1.57	2.39 ± 1.31	1.872	0.166	0.077	0.333	0.567	0.008	1.976	0.167	0.043
Mesomorphy	5.13 ± 1.30	4.52 ± 1.32	4.16 ± 0.97	1.153	0.325	0.049	0.068	0.795	0.002	3.688	0.061	0.077
Ectomorphy	2.42 ± 1.63	3.66 ± 2.84	3.68 ± 1.15	0.825	0.445	0.035	0.135	0.715	0.003	0.054	0.817	0.001
∑6 Skinfolds (mm)	97.24 ± 46.18	61.17 ± 29.14	57.51 ± 27.59	4.368	0.018	0.163	0.456	0.503	0.010	2.920	0.095	0.062
∑8 Skinfolds (mm)	122.38 ± 58.60	77.88 ± 38.45	72.16 ± 36.01	3.976	0.026	0.150	0.381	0.540	0.009	2.619	0.113	0.056
Fat mass percentage (%)	23.12 ± 9.84	15.60 ± 6.12	15.29 ± 8.01	3.802	0.030	0.145	0.916	0.344	0.020	4.566	0.038	0.094
Muscle mass (%)	36.95 ± 2.17	38.92 ± 2.65	38.46 ± 2.30	1.952	0.154	0.080	0.835	0.366	0.019	36.879	<0.001	0.456
Bone mass percentage (%)	16.18 ± 2.78	18.32 ± 2.29	18.66 ± 2.39	2.884	0.066	0.114	1.385	0.246	0.031	21.603	<0.001	0.329
Fat mass (Kg)	19.76 ± 11.41	9.73 ± 4.75	8.18 ± 5.70	8.876	0.001	0.283	0.053	0.819	0.001	0.462	0.500	0.010
Muscle mass (Kg)	29.98 ± 4.73	23.47 ± 3.27	19.34 ± 2.29	18.972	<0.001	0.457	27.716	<0.001	0.386	1.179	0.284	0.026
Bone mass (Kg)	12.88 ± 0.97	10.94 ± 1.15	9.33 ± 1.11	19.015	<0.001	0.458	20.017	<0.001	0.313	1.070	0.307	0.024
BMI (Kg/m2)	24.73 ± 4.50	21.01 ± 3.14	19.10 ± 2.25	6.003	0.005	0.211	1.049	0.311	0.023	4.155	0.048	0.086
Muscle-bone index	2.33 ± 0.37	2.15 ± 0.24	2.08 ± 0.20	2.174	0.126	0.088	2.967	0.092	0.063	5.297	0.026	0.107
Sit & Reach test (cm)	3.01 ± 10.91	0.47 ± 8.50	0.75 ± 6.70	0.276	0.760	0.012	2.248	0.141	0.049	2.008	0.164	0.044
Back scrartch test (cm)	4.76 ± 7.74	0.88 ± 7.47	1.38 ± 5.61	0.914	0.408	0.039	0.165	0.687	0.004	0.306	0.583	0.007
Long jump (m)	1.94 ± 0.25	2.05 ± 0.29	1.86 ± 0.30	1.604	0.212	0.067	18.916	<0.001	0.301	0.378	0.542	0.009
Medicine ball throw (m)	7.58 ± 1.18	6.04 ± 1.25	4.78 ± 0.94	10.191	<0.001	0.312	43.538	<0.001	0.497	10.719	0.002	0.196
CMJ (cm)	0.29 ± 0.05	0.30 ± 0.06	0.26 ± 0.06	1.109	0.339	0.047	10.952	0.002	0.199	0.818	0.371	0.018
CMJ power (W)	942.14 ± 138.85	719.00 ± 129.84	561.29 ± 106.58	16.978	<0.001	0.430	42.958	<0.001	0.494	7.783	0.008	0.150
20 m sprint (s)	3.95 ± 0.27	3.78 ± 0.26	3.88 ± 0.30	1.372	0.264	0.057	8.408	0.006	0.160	103.792	<0.001	0.702
Agility test (s)	8.95 ± 0.56	8.97 ± 0.66	9.32 ± 1.09	0.704	0.500	0.030	9.460	0.004	0.177	88.933	<0.001	0.669

Regarding anthropometric variables, significant differences were observed in basic measurements and BMI (*F* = 6.003–20.828; *p* < 0.001–0.005); in upper limb length (*F* = 9.959; *p* < 0.001); in all bone diameters (*F* = 6.354–18.308; *p* < 0.001–0.004); in all corrected muscle girths (*F* = 11.318–12.759; *p* < 0.001); in the ∑6 and ∑8 skinfolds and fat mass in kg and percentage (*F* = 8.876–4.368; *p* < 0.001–0.030); and in muscle and bone masses in kg (*F* = 18.972–19.015; *p* < 0.001). The inclusion of the covariable “age” showed significant effect in the model in the same variables (*F* = 4.469–88.401; *p* < 0.001–0.040), except in the ∑6 and ∑8 skinfolds, in the fat percentage and in the bone mass (Kg). The interaction between variables showed that differences between maturity groups were influenced by age in the bone related variables (*F* = 24.211–91.052; *p* < 0.001), in the muscle related variables (*F* = 5.248–22.974; *p* < 0.001–0.027) and in the percentages of body composition (*F* = 4.566–36.879; *p* < 0.001–0.038).

Pairwise comparisons after Bonferroni adjustment regarding the anthropometric variables can be seen in Tables 2 and 3. The early maturers group obtained higher values in the anthropometric variables than the average and late maturers (Tables 2 and 3), showing significant differences in all anthropometric variables (*p* < 0.001–0.030), except in the ∑6 and ∑8 skinfolds and in the fat mass percentage, where differences were found only between the early and average maturers (*p* = 0.020–0.030). The interaction maturity group*age showed that age had a significant influence in the pairwise differences in all anthropometric variables (*p* < 0.001–0.037).

**Table 2:** *Post hoc* comparison between groups with significant differences in the ANCOVA analysis for the maturational status variables and bone-related kinanthropometric variables.

Test	Groups comparation		Model					
			Maturation group			Maturation group × Age		
			Mean difference ± SD	*p* value	95% CI	Mean difference ± SD	*p* value	95% CI
Maturity offset (years)	E	A	1.16 ± 0.38	0.012	0.21 to 2.11	0.80 ± 0.12	<0.001	0.50 to 1.09
		E	L	1.55 ± 0.50	0.010	0.30 to 2.80	1.69 ± 0.16	<0.001	1.30 to 2.08
	A	L	0.39 ± 0.40	1.000	−0.61 to 1.39	0.89 ± 0.13	<0.001	0.58 to 1.21
APHV (years)	E	A	−0.76 ± 0.12	<0.001	−1.07 to −0.46	−0.80 ± 0.12	<0.001	−1.09 to −0.50
		E	L	−1.70 ± 0.16	<0.001	−2.09 to −1.30	−1.69 ± 0.16	<0.001	−2.07 to −1.30
	A	L	−0.93 ± 0.13	<0.001	−1.25 to −0.62	−0.89 ± 0.13	<0.001	−1.21 to -0.58
Body mass (Kg)	E	A	20.86 ± 3.84	<0.001	11.29 to 30.42	18.92 ± 3.39	<0.001	10.40 to 27.36
		E	L	30.75 ± 5.05	<0.001	18.19 to 43.31	31.50 ± 4.41	<0.001	20.52 to 42.48
	A	L	9.89 ± 4.06	0.057	−0.21 to 19.99	12.58 ± 3.61	0.003	3.59 to 21.56
Height (cm)	E	A	11.72 ± 2.97	0.001	4.34 to 19.11	9.91 ± 2.40	<0.001	3.94 to 15.89
		E	L	19.09 ± 3.90	<0.001	9.39 to 28.79	19.79 ± 3.12	<0.001	12.02 to 27.57
	A	L	7.36 ± 3.13	0.070	−0.43 to 15.16	9.87 ± 2.55	0.001	3.51 to 16.24
Arm span (cm)	E	A	12.91 ± 3.31	0.001	4.69 to 21.14	11.30 ± 2.95	0.001	3.96 to 18.64
		E	L	20.78 ± 4.34	<0.001	9.98 to 31.58	21.40 ± 3.83	<0.001	11.86 to 30.95
	A	L	7.86 ± 3.49	0.088	−0.82 to 16.54	10.10 ± 3.14	0.007	2.29 to 17.91
Sitting height (cm)	E	A	6.00 ± 1.50	0.001	2.26 to 9.73	4.77 ± 0.88	<0.001	2.57 to 6.98
		E	L	10.27 ± 1.97	<0.001	5.37 to 15.81	10.75 ± 1.15	<0.001	7.89 to 13.62
	A	L	4.27 ± 1.58	0.029	0.33 to 8.22	5.97 ± 0.94	<0.001	3.63 to 8.32
Upper limb length (cm)	E	A	5.50 ± 1.45	0.001	1.89 to 9.10	4.83 ± 1.31	0.002	1.56 to 8.11
		E	L	7.96 ± 1.90	<0.001	3.22 to 12.70	8.22 ± 1.71	<0.001	3.96 to 12.48
	A	L	2.46 ± 1.53	0.344	−1.34 to 6.27	3.38 ± 1.40	0.060	−0.10 to 6.87
Iliospinale height (cm)	E	A	8.08 ± 5.11	0.362	−4.62 to 20.79	6.10 ± 4.81	0.635	−5.88 to 18.08
		E	L	16.00 ± 6.71	0.064	−0.69 to 32.69	16.77 ± 6.26	0.031	1.20 to 32.35
	A	L	7.91 ± 5.39	0.448	−5.50 to 21.34	10.67 ± 5.12	0.129	−2.08 to 23.42
Biacromial breadth (cm)	E	A	3.99 ± 0.84	<0.001	1.90 to 6.08	3.45 ± 0.66	<0.001	1.82 to 5.10
		E	L	5.51 ± 1.10	<0.001	2.76 to 8.25	5.71 ± 0.86	<0.001	3.58 to 7.85
	A	L	1.51 ± 0.89	0.285	−0.69 to 3.72	2.26 ± 0.70	0.007	0.52 to 4.01
Biiliocrestal breadth (cm)	E	A	3.40 ± 0.63	<0.001	1.83 to 4.97	3.08 ± 0.55	<0.001	1.70 to 4.45
		E	L	4.53 ± 0.83	<0.001	2.47 to 6.58	4.65 ± 0.72	<0.001	2.87 to 6.44
	A	L	1.12 ± 0.67	0.293	−0.53 to 2.78	1.58 ± 0.59	0.031	0.11 to 3.04
Femur breadth (cm)	E	A	0.70 ± 0.17	<0.001	0.28 to 1.12	0.67 ±’.17	0.001	0.24 to 1.09
		E	L	1.27 ± 0.22	<0.001	0.72 to 1.82	1.28 ± 0.22	<0.001	0.73 to 1.83
	A	L	0.57 ± 0.18	0.007	0.13 to 1.01	0.62 ± 0.18	0.004	0.17 to 1.07
Humerus breadth (cm)	E	A	0.43 ± 0.14	0.010	0.08 to 0.78	0.37 ± 0.13	0.018	0.05 to 0.69
		E	L	0.71 ± 0.18	0.001	0.25 to 1.16	0.72 ± 0.17	<0.001	0.31 to 1.15
	A	L	0.27 ± 0.15	0.212	−0.09 to 0.64	0.36 ± 0.14	0.037	0.02 to 0.70
Bi-styloid breadth (cm)	E	A	0.16 ± 0.12	0.608	−0.14 to 0.46	0.12 ± 0.12	0.945	−0.17 to 0.41
		E	L	0.55 ± 0.16	0.004	0.15 to 0.95	0.56 ± 0.15	0.002	0.18 to 0.95
	A	L	0.39 ± 0.13	0.012	0.07 to 0.71	0.44 ± 0.13	0.003	0.13 to 0.76

**Notes.**

E Early maturers A Average maturers L Late maturers

**Table 3:** *Post hoc* comparison between groups with significant differences in the ANCOVA analysis for the muscle- and fat-related kinanthropometric variables, and body composition variables.

Test	Groups comparation		Model					
			Maturation group			Maturation group × Age		
			Mean difference ± SD	*p* value	95% CI	Mean difference ±SD	*p* value	95% CI
Corrected arm girth (cm)	E	A	3.65 ± 0.92	0.001	1.36 to 5.94	3.31 ± 0.87	0.001	1.14 to 5.49
		E	L	5.93 ± 1.21	<0.001	2.92 to 8.94	6.10 ± 1.14	<0.001	3.23 to 8.90
	A	L	2.28 ± 0.97	0.071	−0.14 to 4.70	2.75 ± 0.93	0.015	0.43 to 5.07
Corrected thigh girth (cm)	E	A	4.72 ± 1.46	0.007	1.09 to 8.35	4.06 ± 1.33	0.011	0.75 to 7.37
		E	L	9.31 ± 1.91	<0.001	4.54 to 14.08	9.57 ± 1.73	<0.001	5.26 to 13.87
	A	L	4.59 ± 1.54	0.014	0.75 to 8.42	5.51 ± 1.73	0.001	1.98 to 9.03
Corrected leg girth (cm)	E	A	2.59 ± 0.75	0.004	0.71 to 4.45	2.16 ± 0.63	0.004	0.58 to 3.74
		E	L	4.65 ± 0.99	<0.001	2.19 to 7.10	4.81 ± 0.82	<0.001	2.76 to 6.87
	A	L	2.06 ± 0.79	0.038	0.09 to 4.03	2.65 ± 0.68	0.001	0.97 to 4.33
∑6 Skinfolds (mm)	E	A	36.07 ± 12.69	0.020	4.50 to 67.62	37.34 ± 12.91	0.018	5.21 to 69.47
		E	L	39.73 ± 16.67	0.064	−1.71 to 81.18	39.23 ± 16.78	0.072	−2.54 to 81.08
	A	L	3.67 ± 13.40	1.000	−29.65 to 36.99	1.89 ± 13.74	1.000	−32.30 to 36.08
∑8 Skinfolds (mm)	E	A	44.48 ± 16.52	0.030	3.39 to 85.58	46.01 ± 16.82	0.027	4.14 to 87.88
		E	L	50.21 ± 21.70	0.076	−3.75 to 104.18	49.62 ± 21.87	0.085	−4.82 to 104.07
	A	L	5.73 ± 17.45	1.000	−37.66 to 49.12	3.61 ± 17.09	1.000	−40.95 to 48.17
Fat mass percentage (%)	E	A	7.51 ± 2.79	0.030	0.56 to 14.47	7.91 ± 2.83	0.023	0.87 to 14.96
		E	L	7.82 ± 3.67	0.116	−1.31 to 16.95	7.67 ± 3.68	0.129	−1.49 to 16.82
	A	L	0.30 ± 2.95	1.000	−7.03 to 7.64	−0.25 ± 3.01	1.000	−7.74 to 7.24
Fat mass (Kg)	E	A	10.03 ± 2.51	0.001	3.78 to 16.28	9.95 ± 2.57	0.001	3.56 to 16.34
		E	L	11.58 ± 3.30	0.003	3.37 to 19.78	11.61 ± 3.34	0.003	3.31 to 19.92
	A	L	1.55 ± 2.65	1.000	−5.05 to 8.14	1.67 ± 2.73	1.000	−5.13 to 8.47
Muscle mass (Kg)	E	A	6.51 ± 1.35	<0.001	3.14 to 9.88	5.67 ± 1.08	<0.001	2.97 to 8.37
		E	L	10.64 ± 1.78	<0.001	6.22 to 15.07	10.97 ± 1.41	<0.001	7.46 to 14.48
	A	L	4.13 ± 1.43	0.018	0.58 to 7.69	5.30 ± 1.15	<0.001	2.43 to 8.17
Bone mass (Kg)	E	A	1.93 ± 0.44	<0.001	0.84 to 3.03	1.69 ± 0.37	<0.001	0.76 to 2.62
		E	L	3.54 ± 0.58	<0.001	2.10 to 4.98	3.64 ± 0.49	<0.001	2.43 to 4.85
	A	L	1.61 ± 0.46	0.001	0.45 to 2.76	1.95 ± 0.40	<0.001	0.96 to 2.94
BMI (Kg/m2)	E	A	3.72 ± 1.30	0.019	0.48 to 6.95	3.52 ± 1.31	0.031	0.25 to 6.79
		E	L	5.63 ± 1.71	0.006	1.38 to 9.87	5.71 ± 1.71	0.005	1.46 to 9.96
	A	L	1.91 ± 1.37	0.509	−1.50 to 5.32	2.19 ± 1.40	0.373	−1.29 to 5.67

**Notes.**

E Early maturers A Average maturers L Late maturers

In the physical fitness tests (Table 1), the ANOVA identified significant differences between groups in the medicine ball throw (*F* = 10.191; *p* < 0.001) and in CMJ power (*F* = 16.978; *p* < 0.001). The inclusion in the ANCOVA of the covariate age showed, in addition to the previous tests, effect in the long jump, CMJ height, sprint and agility (*F* = 8.408–43.538; *p* < 0.001–0.006), while the interaction maturity group*age showed significant influence of the age in the differences found between groups in the medicine ball throw, CMJ power, sprint and agility (*F* = 10.719–103.792; *p* < 0.001–0.008).

The significant differences found after Bonferroni adjustment in the physical tests are shown in Table 4. In the medicine ball throw test and the CMJ power test, the early maturers group obtained better results than the average and late maturers groups (*p* < 0.001–0.007); while the average group obtained better results than the late maturers group (*p* = 0.016–0.047). The same differences between groups were found in these tests when age was included as a covariate in the model (*p* < 0.001–0.004).

**Table 4:** *Post hoc* comparison between groups with significant differences in the ANCOVA analysis for the physical fitness variables.

Test	Groups comparation		Model					
			Maturation group			Maturation group*Age		
			Mean difference ± SD	*p* value	95% CI	Mean difference ± SD	*p* value	95% CI
Medicine ball throw (m)	E	A	1.53 ± 0.48	0.007	0.35 to 2.72	1.20 ± 0.34	0.004	0.34 to 2.06
		E	L	2.80 ± 0.62	<0.001	1.24 to 4.35	2.93 ± 0.45	<0.001	1.81 to 4.05
	A	L	1.26 ± 0.50	0.047	0.014 to 2.51	1.73 ± 0.36	<0.001	0.81 to 2.64
CMJ power (W)	E	A	223.14 ± 50.62	<0.001	97.24 to 349.03	187.67 ± 36.81	<0.001	96.03 to 279. 31
		E	L	379.85 ± 66.48	<0.001	214.51 to 545.18	393.63 ± 47.87	<0.001	274.48 to 512.79
	A	L	156.71 ± 53.45	0.016	23.77 to 289.65	205.96 ± 39.18	<0.001	108.44 to 303.49

**Notes.**

E Early maturers A Average maturers L Late maturers

Tables 5 and 6 show the correlations between anthropometric variables and physical performance variables. Both maturity offset and age showed moderate to high correlations with the physical fitness test (*r* = 0.238–0.810; *p* < 0.001–0.021). The horizontal jump test and the CMJ showed moderate positive correlations with height, sitting height, bi-styloid breath, corrected leg girth and muscle mass percentage (*r* = 0.301–0.462; *p* < 0.001–0.038); and moderate negative correlations with both fat mass and fat percentage (*r* =  − 0.427, −0.511; *p* < 0.001–0.002). Medicine ball throw showed moderate to high positive correlations with all variables (*r* = 0.330–0.829; *p* < 0.001–0.022), except with ∑8 skinfolds, fat mass percentage and muscle mass percentage. CMJ power showed moderate to high positive correlations with all anthropometric variables analysed, except for muscle mass percentage (*r* = 0.367–0.921; *p* < 0.001–0.010). Sprint time showed moderate positive correlations with the fat-related variables, BMI and with the musculoskeletal index (*r* = 0.400–0.670; *p* < 0.001–0.005), while the correlation with muscle mass percentage was moderate negative (*r* =  − 0.459; *p* = 0.001). The agility test showed moderate and low negative correlations with the variables height, arm span, sitting height, upper limb length, iliospinale height, biacromial breadth and corrected leg circumference (*r* =  − 0.286, −0.488; *p* < 0.001–0.049), while with fat mass variables the correlation was moderate positive (*r* = 0.333–0.357; *p* = 0.013–0.021).

**Table 5:** Correlations between anthropometric and physical fitness variables.

	Sit & reach	Back scratch test	Long jump	Medicine ball throw	CMJ	CMJ power	20 m sprint	Agility test
Maturity offset	*r* = 0.282; *p* = 0.052	*r* = 0.065; *p* = 0.662	*r* = 0.429; *p* = 0.002	*r* = 0.810; *p* < 0.001	*r* = 0.366; *p* = 0.011	*r* = 0.808; *p* < 0.001	*r* = − 0.238; *p* = 0.104	*r* = − 0.402; *p* = 0.005
Age	*r* = 0.228; *p* = 0.119	*r* = − 0.032; *p* = 0.829	*r* = 0.460; *p* = 0.001	*r* = 0.563; *p* < 0.001	*r* = 0.380; *p* = 0.008	*r* = 0.518; *p* < 0.001	r=-0.333; *p* = 0.021	*r* = − 0.376; *p* = 0.008
Body mass	*r* = 0.160; *p* = 0.278	*r* = 0.147; *p* = 0.318	*r* = 0.017; *p* = 0.906	*r* = 0.660; *p* < 0.001	*r* = − 0.018; *p* = 0.904	*r* = 0.888; *p* < 0.001	*r* = 0.259; *p* = 0.075	*r* = − 0.062; *p* = 0.678
Height	*r* = 0.144; *p* = 0.329	*r* = 0.174; *p* = 0.237	*r* = 0.330; *p* = 0.022	*r* = 0.690; *p* < 0.001	*r* = 0.376; *p* = 0.008	*r* = 0.723; *p* < 0.001	*r* = − 0.142; *p* = 0.336	*r* = − 0.337; *p* = 0.019
Arm span	*r* = 0.240; *p* = 0.101	*r* = 0.274; *p* = 0.060	*r* = 0.234; *p* = 0.110	*r* = 0.635; *p* < 0.001	*r* = 0.267; *p* = 0.066	*r* = 0.706; *p* < 0.001	*r* = − 0.033; *p* = 0.823	*r* = − 0.317; *p* = 0.028
Sitting height	*r* = 0.279; *p* = 0.054	*r* = 0.103; *p* = 0.485	*r* = 0.407; *p* = 0.004	*r* = 0.829; *p* < 0.001	*r* = 0.365; *p* = 0.011	*r* = 0.795; *p* < 0.001	*r* = − 0.233; *p* = 0.111	*r* = − 0.420; *p* = 0.003
Upper limb length	*r* = 0.191; *p* = 0.194	*r* = 0.267; *p* = 0.066	*r* = 0.248; *p* = 0.090	*r* = 0.596; *p* < 0.001	*r* = 0.263; *p* = 0.071	*r* = 0.695; *p* < 0.001	*r* = − 0.045; *p* = 0.762	*r* = − 0.300; *p* = 0.038
Iliospinale height	*r* = 0.172; *p* = 0.241	*r* = 0.155; *p* = 0.292	*r* = 0.224; *p* = 0.125	*r* = 0.414; *p* = 0.003	*r* = 0.180; *p* = 0.220	*r* = 0.393; *p* = 0.006	*r* = 0.043; *p* = 0.772	*r* = − 0.488; *p* < 0.001
Biacromial breadth	*r* = 0.225; *p* = 0.124	*r* = 0.212; *p* = 0.148	*r* = 0.258; *p* = 0.077	*r* = 0.736; *p* < 0.001	*r* = 0.234; *p* = 0.109	*r* = 0.806; *p* < 0.001	*r* = − 0.037; *p* = 0.803	*r* = − 0.286; *p* = 0.049
Biiliocrestal breadth	*r* = 0.193; *p* = 0.189	*r* = 0.189; *p* = 0.198	*r* = 0.084; *p* = 0.570	*r* = 0.629; *p* < 0.001	*r* = 0.050; *p* = 0.734	*r* = 0.795; *p* < 0.001	*r* = 0.197; *p* = 0.179	*r* = − 0.106; *p* = 0.473
Femur breadth	*r* = 0.122; *p* = 0.409	*r* = − 0.008; *p* = 0.956	*r* = 0.029; *p* = 0.843	*r* = 0.503; *p* < 0.001	*r* = 0.027; *p* = 0.854	*r* = 0.677; *p* < 0.001	*r* = 0.062; *p* = 0.674	*r* = 0.036; *p* = 0.808
Humerus breadth	*r* = 0.151; *p* = 0.306	*r* = 0.175; *p* = 0.235	*r* = 0.235; *p* = 0.108	*r* = 0.504; *p* < 0.001	*r* = 0.043; *p* = 0.774	*r* = 0.595; *p* < 0.001	*r* = − 0.064; *p* = 0.665	*r* = − 0.192; *p* = 0.190
Bi-styloid breatdth	*r* = 0.063; *p* = 0.671	*r* = − 0.114; *p* = 0.439	*r* = 0.420; *p* = 0.003	*r* = 0.407; *p* = 0.004	*r* = 0.301; *p* = 0.038	*r* = 0.507; *p* < 0.001	*r* = − 0.302; *p* = 0.037	*r* = − 0.264; *p* = 0.070
Corrected arm girth	*r* = 0.254; *p* = 0.082	*r* = 0.034; *p* = 0.816	*r* = 0.129; *p* = 0.383	*r* = 0.656; *p* < 0.001	*r* = 0.067; *p* = 0.651	*r* = 0.792; *p* < 0.001	*r* = 0.120; *p* = 0.415	*r* = − 0.104; *p* = 0.480
Corrected thigh girth	*r* = 0.115; *p* = 0.438	*r* = 0.077; *p* = 0.605	*r* = 0.109; *p* = 0.461	*r* = 0.623; *p* < 0.001	*r* = 0.061; *p* = 0.681	*r* = 0.849; *p* < 0.001	*r* = 0.192; *p* = 0.191	*r* = − 0.040; *p* = 0.785
Corrected leg girth	*r* = 0.305; *p* = 0.035	*r* = 0.005; *p* = 0.973	*r* = 0.356; *p* = 0.013	*r* = 0.687; *p* < 0.001	*r* = 0.370; *p* = 0.010	*r* = 0.820; *p* < 0.001	*r* = − 0.058; *p* = 0.696	*r* = − 0.296; *p* = 0.041
BMI	*r* = 0.130; *p* = 0.380	*r* = 0.084; *p* = 0.571	*r* = − 0.164; *p* = 0.267	*r* = 0.384; *p* = 0.007	*r* = − 0.245; *p* = 0.093	*r* = 0.648; *p* < 0.001	*r* = 0.400; *p* = 0.005	*r* = 0.113; *p* = 0.445
∑8 Skinfolds	*r* = 0.067; *p* = 0.653	*r* = 0.081; *p* = 0.584	*r* = − 0.427; *p* = 0.002	*r* = 0.170; *p* = 0.249	*r* = − 0.489; *p* < 0.001	*r* = 0.417; *p* = 0.003	*r* = 0.611; *p* < 0.001	*r* = 0.333; *p* = 0.021
Fat mass (%)	*r* = 0.071; *p* = 0.630	*r* = 0.131; *p* = 0.377	*r* = − 0.474; *p* = 0.001	*r* = 0.137; *p* = 0.354	*r* = − 0.511; *p* < 0.001	*r* = 0.367; *p* = 0.010	*r* = 0.670; *p* < 0.001	*r* = 0.357; *p* = 0.013
Muscle mass (%)	*r* = − 0.005; *p* = 0.971	*r* = − 0.276; *p* = 0.057	*r* = 0.424; *p* = 0.003	*r* = 0.025; *p* = 0.864	*r* = 0.462; *p* = 0.001	*r* = − 0.174; *p* = 0.237	*r* = − 0.459; *p* = 0.001	*r* = − 0.171; *p* = 0.245
Fat mass (Kg)	*r* = 0.093; *p* = 0.528	*r* = 0.104; *p* = 0.480	*r* = − 0.354; *p* = 0.014	*r* = 0.330; *p* = 0.022	*r* = − 0.379; *p* = 0.008	*r* = 0.564; *p* < 0.001	*r* = 0.590; *p* < 0.001	*r* = 0.240; *p* = 0.100
Muscle mass (Kg)	*r* = 0.179; *p* = 0.223	*r* = 0.076; *p* = 0.606	*r* = 0.188; *p* = 0.202	*r* = 0.747; *p* < 0.001	*r* = 0.160; *p* = 0.279	*r* = 0.921; *p* < 0.001	*r* = 0.110; *p* = 0.458	*r* = − 0.145; *p* = 0.325
Muscle-bone index	*r* = 0.152; *p* = 0.303	*r* = 0.073; *p* = 0.621	r=-0.058; *p* = 0.694	*r* = 0.405; *p* = 0.004	*r* = − 0.089; *p* = 0.547	*r* = 0.555; *p* < 0.001	*r* = 0.322; *p* = 0.026	*r* = 0.048; *p* = 0.744

**Table 6:** Correlations between muscle-related, fat-related kinanthropometric variables and physical fitness variables.

	Long jump	Medicine ball throw	CMJ	CMJ power	20 m sprint	Agility test
Corrected arm girth	*r* = 0.129; *p* = 0.383	*r* = 0.656; *p* < 0.001	*r* = 0.067; *p* = 0.651	*r* = 0.792; *p* < 0.001	*r* = 0.120; *p* = 0.415	*r* = − 0.104; *p* = 0.480
Corrected thigh girth	*r* = 0.109; *p* = 0.461	*r* = 0.623; *p* < 0.001	*r* = 0.061; *p* = 0.681	*r* = 0.849; *p* < 0.001	*r* = 0.192; *p* = 0.191	*r* = − 0.040; *p* = 0.785
Corrected leg girth	*r* = 0.356; *p* = 0.013	*r* = 0.687; *p* < 0.001	*r* = 0.370; *p* = 0.010	*r* = 0.820; *p* < 0.001	*r* = − 0.058; *p* = 0.696	*r* = − 0.296; *p* = 0.041
BMI	*r* = − 0.164; *p* = 0.267	*r* = 0.384; *p* = 0.007	*r* = − 0.245; *p* = 0.093	*r* = 0.648; *p* < 0.001	*r* = 0.400; *p* = 0.005	*r* = 0.113; *p* = 0.445
∑8 Skinfolds	*r* = − 0.427; *p* = 0.002	*r* = 0.170; *p* = 0.249	*r* = − 0.489; *p* < 0.001	*r* = 0.417; *p* = 0.003	*r* = 0.611; *p* < 0.001	*r* = 0.333; *p* = 0.021
Fat mass (%)	*r* = − 0.474; *p* = 0.001	*r* = 0.137; *p* = 0.354	*r* = − 0.511; *p* < 0.001	*r* = 0.367; *p* = 0.010	*r* = 0.670; *p* < 0.001	*r* = 0.357; *p* = 0.013
Muscle mass (%)	*r* = 0.424; *p* = 0.003	*r* = 0.025; *p* = 0.864	*r* = 0.462; *p* = 0.001	*r* = − 0.174; *p* = 0.237	*r* = − 0.459; *p* = 0.001	*r* = − 0.171; *p* = 0.245
Fat mass (Kg)	*r* = − 0.354; *p* = 0.014	*r* = 0.330; *p* = 0.022	*r* = − 0.379; *p* = 0.008	*r* = 0.564; *p* < 0.001	*r* = 0.590; *p* < 0.001	*r* = 0.240; *p* = 0.100
Muscle mass (Kg)	*r* = 0.188; *p* = 0.202	*r* = 0.747; *p* < 0.001	*r* = 0.160; *p* = 0.279	*r* = 0.921; *p* < 0.001	*r* = 0.110; *p* = 0.458	*r* = − 0.145; *p* = 0.325
Muscle-bone index	*r* = − 0.058; *p* = 0.694	*r* = 0.405; *p* = 0.004	*r* = − 0.089; *p* = 0.547	*r* = 0.555; *p* < 0.001	*r* = 0.322; *p* = 0.026	*r* = 0.048; *p* = 0.744

Table 7 shows the linear regression models in relation to the fitness tests, as well as the resulting predictive equations for each physical fitness test. Between one and three prediction models were found for the performance in the different fitness tests, which could explain 47 to 88% of the cases based on the anthropometric variables (*p* < 0.001). The most determinant anthropometric variables were height, sitting height, iliospinale height, the arm and calf corrected girths, muscle mass (kg) and fat mass percentage.

**Table 7:** Regression models of the performance in the different physical fitness tests.

Variable	Analysis	R^2^	*p* value	Included independent variables	SC	*p* value	Equation
Long jump	Model 1	0.47	0.001	Fat mass (%)	−0.47	<0.001	Long jump = 2.322 − 0.019*fat mass (%)
		Model 2	0.68	<0.001	Fat mass (%)	−0.53	<0.001	Long jump = 2.277 − 0.021*fat mass (%) + 0.135*maturity offset (years)
	Maturity offset				0.49	<0.001		
Medicine ball throw	Model 1	0.69	<0.001	Sitting height	0.83	<0.001	Medicine ball throw = − 15.667 + 0.249*sitting height
		Model 2	0.74	<0.001	Sitting height	0.67	<0.001	Medicine ball throw = −14.710 + 0.202*sitting height + 0.136*corrected arm girth
	Corrected arm girth				0.27	0.005		
CMJ	Model 1	0.26	<0.001	Fat mass (%)	−0.51	<0.001	CMJ = 36.625 − 0.421*fat mass (%)
		Model 2	0.53	<0.001	Fat mass (%)	−0.65	<0.001	CMJ = −7.860 − 0.538*fat mass (%) + 1.462*corrected leg girth
	Corrected leg girth				0.54	<0.001		
CMJ power	Model 1	0.85	<0.001	Muscle mass	0.92	<0.001	CMJ power = −71.982 + 33.613*muscle mass (kg)
		Model 2	0.88	<0.001	Muscle mass	0.77	<0.001	CMJ power = −689.738 + 28.077*muscle mass (kg) + 4.396*height (cm)
	Height				0.24	<0.001		
20 m sprint	Model 1	0.45	<0.001	Fat mass (%)	0.67	<0.001	Sprint = 3.425 + 0.024*fat mass (%)
		Model 2	0.53	<0.001	Fat mass (%)	0.65	<0.001	Sprint = 4.488 + 0.023*fat mass (%) − 0.074*age (years)
	Age				−0.28	<0.001		
Agility test	Model 1	0.45	<0.001	Fat mass (%)	0.67	<0.001	Agility = 3.425 + 0.024*fat mass (%)
		Model 2	0.55	<0.001	Fat mass (%)	0.70	<0.001	Agility = 3.452 + 0.025*fat mass (%) − 0.080*maturity offset (years)
					Maturation offset	−0.32	0.004	
		Model 3	0.60	<0.001	Fat mass (%)	0.72	0.001	Agility = 2.951 + 0.026*fat mass (%) − 0.115*maturity offset (years) + 0.005*ilioespinale height (cm)
									Maturation offset	−0.46	0.001	
	Ilioespinale height				0.27	0.017		

**Notes.**

SC Standardiced coeficients

Tables 8 to 11 show the correlations between anthropometric variables and physical performance variables dividing the sample in age groups. It was observed that in the 12-years-old group (Table 8), the anthropometric variables were related to sport performance only in CMJ power, showing positive high correlations with the body mass and BMI, corrected girths, ∑8 Skinfolds, fat and muscle masses (kg) and muscle-bone index (*r* = 0.786–0.934; *p* = 0.002–0.036).

**Table 8:** Correlations between anthropometric and fitness variables in 12 years old group.

12 years (*n* = 7)	CMJ	CMJ power
Maturity offset	*r* = − 0.760; *p* = 0.047	–
Body mass	–	*r* = 0.877; *p* = 0.010
Corrected arm girth	–	*r* = 0.898; *p* = 0.006
Corrected thigh girth	–	*r* = 0.868; *p* = 0.011
Corrected leg girth	–	*r* = 0.787; *p* = 0.036
BMI	–	*r* = 0.934; *p* = 0.002
∑8 Skinfolds	–	*r* = 0.815; *p* = 0.026
Fat mass (Kg)	–	*r* = 0.801; *p* = 0.030
Muscle mass (Kg)	–	*r* = 0.909; *p* = 0.005
Muscle-bone index	–	*r* = 0.786; *p* = 0.036

**Table 9:** Correlations between anthropometric and fitness variables in 13 years old group.

13 years (*n* = 15)	Long jump	Medicine ball throw	CMJ	CMJ power	20 m sprint	Agility test
Maturity offset	*r* = 0.604; *p* = 0.017	*r* = 0.792; *p* < 0.001	–	*r* = 0.656; *p* = 0.008	–	–
Body mass	–	*r* = 0.617; *p* = 0.014	–	*r* = 0.855; *p* < 0.001	–	–
Height	–	*r* = 0.647; *p* = 0.009	–	*r* = 0.521; *p* = 0.046	–	–
Arm spam	–	*r* = 0.620; *p* = 0.014	–	*r* = 0.609; *p* = 0.016	–	*r* = − 0.551; *p* = 0.033
Sitting height	*r* = 0.686; *p* = 0.005	*r* = 0.702; *p* = 0.004	–	*r* = 0.586; *p* = 0.022	–	*r* = − 0.537; *p* = 0.039
Upper limb length	–	*r* = 0.554; *p* = 0.036	–	*r* = 0.671; *p* = 0.006	–	*r* = − 0.300; *p* = 0.038
Biacromial breadth	–	–	–	*r* = 0.648; *p* = 0.009	–	–
Biiliocrestal breadth	–	*r* = 0.576; *p* = 0.024	–	*r* = 0.747; *p* < 0.001	–	–
Femur breadth	–	*r* = 0.579; *p* = 0.024	–	*r* = 0.828; *p* < 0.001	–	–
Humerus breadth	–	*r* = 0.566; *p* = 0.028	–	*r* = 0.672; *p* = 0.006	–	–
Bi-styloid breadth	*r* = 0.534; *p* = 0.040	–	–	–	–	*r* = − 0.555; *p* = 0.032
Corrected arm girth	–	–	–	*r* = 0.631; *p* = 0.012	–	–
Corrected thigh girth	–	–	–	*r* = 0.759; *p* = 0.001	*r* = 0.550; *p* = 0.034	–
Corrected leg girth	–	*r* = 0.578; *p* = 0.024	–	*r* = 0.723; *p* = 0.002	–	–
BMI	–	–	*r* = − 0.544; *p* = 0.036	*r* = 0.583; *p* = 0.023	*r* = 0.527; *p* = 0.043	–
∑8 Skinfolds	*r* = − 0.427; *p* = 0.002	–	*r* = − 0.594; *p* = 0.020	–	*r* = 0.608; *p* = 0.016	–
Fat mass (%)	*r* = − 0.522; *p* = 0.046	–	*r* = − 0.561; *p* = 0.030	–	*r* = 0.621; *p* = 0.013	–
Fat mass (Kg)	–	–	–	*r* = 0.613; *p* = 0.012	*r* = 0.578; *p* = 0.024	–
Muscle mass (Kg)	–	*r* = 0.595; *p* = 0.019	–	*r* = 0.843; *p* < 0.001	–	–
Muscle-bone index	–	–	*r* = − 0.516; *p* = 0.049	–	*r* = 0.625; *p* = 0.009	–

**Table 10:** Correlations between anthropometric and fitness variables in 14 years old group.

14 years (*n* = 12)	Medicine ball throw	CMJ	CMJ power	20 m sprint	Agility test
Maturity offset	*r* = 0.602; *p* = 0.039	–	*r* = 0.766; *p* = 0.004	–	*r* = − 0.653; *p* = 0.021
Body mass	–	–	*r* = 0.943; *p* < 0.001	*r* = 0.736; *p* = 0.006	*r* = 0.720; *p* = 0.008
Height	–	–	*r* = 0.620; *p* = 0.031	–	–
Arm spam	–	–	*r* = 0.603; *p* = 0.038	–	–
Sitting height	*r* = 0.669; *p* = 0.017	–	*r* = 0.617; *p* = 0.033	–	–
Upper limb length	–	–	*r* = 0.695; *p* < 0.001	–	–
Biiliocrestal breadth	–	–	*r* = 0.847; *p* < 0.001	–	*r* = 0.674; *p* = 0.016
Femur breadth	–	–	*r* = 0.626; *p* = 0.029	–	*r* = 0.761; *p* = 0.004
Corrected arm girth	–	–	*r* = 0.833; *p* < 0.001	–	*r* = 0.778; *p* = 0.003
Corrected thigh girth	–	–	*r* = 0.869; *p* < 0.001	*r* = 0.607; *p* = 0.036	*r* = 0.718; *p* = 0.009
Corrected leg girth	–	–	*r* = 0.760; *p* = 0.004	–	–
BMI	–	–	*r* = 0.860; *p* < 0.001	*r* = 0.678; *p* = 0.015	*r* = 0.639; *p* = 0.035
∑8 Skinfolds	–	*r* = − 0.644; *p* = 0.024	*r* = 0.729; *p* = 0.007	*r* = 0.812; *p* = 0.001	*r* = 0.631; *p* = 0.028
Fat mass (%)	–	–	*r* = 0.772; *p* = 0.003	*r* = 0.882; *p* < 0.001	*r* = 0.625; *p* = 0.030
Fat mass (Kg)	–	–	*r* = 0.790; *p* = 0.002	*r* = 0.883; *p* < 0.001	*r* = 0.630; *p* = 0.028
Muscle mass (Kg)	–	–	*r* = 0.901; *p* < 0.001	*r* = 0.640; *p* = 0.025	*r* = − 0.768; *p* = 0.004
Muscle-bone index	–	–	*r* = 0.747; *p* = 0.005	–	–

**Table 11:** Correlations between anthropometric and fitness variables in 15 years old group.

15 years (*n* = 14)	Long jump	Medicine ball throw	CMJ	CMJ power	20 m sprint
Maturity offset	–	*r* = 0.886; *p* < 0.001	–	*r* = 0.915; *p* < 0.001	–
Body mass	–	*r* = 0.730; *p* = 0.003	–	*r* = 0.844; *p* < 0.001	–
Height	–	*r* = 0.736; *p* = 0.003	–	*r* = 0.829; *p* < 0.001	–
Arm spam	–	*r* = 0.735; *p* = 0.003	–	*r* = 0.775; *p* < 0.001	–
Sitting height	–	*r* = 0.909; *p* < 0.001	–	*r* = 0.915; *p* < 0.001	–
Upper limb length	–	*r* = 0.649; *p* = 0.012	–	*r* = 0.757; *p* = 0.002	–
Biacromial breadth	–	*r* = 0.784; *p* < 0.001	–	*r* = 0.933; *p* < 0.001	–
Biiliocrestal breadth	–	*r* = 0.818; *p* < 0.001	–	*r* = 0.820; *p* < 0.001	–
Femur breadth	–	*r* = 0.787; *p* < 0.001	–	*r* = 0.856; *p* < 0.001	–
Humerus breadth	–	*r* = 0.545; *p* = 0.044	–	*r* = 0.567; *p* = 0.035	–
Bi-styloid breatdth	–	*r* = 0.625; *p* = 0.017	–	*r* = 0.835; *p* < 0.001	–
Corrected arm girth	–	*r* = 0.741; *p* = 0.022	–	r=0.798; *p* < 0.001	–
Corrected thigh girth	–	*r* = 0.796; *p* < 0.001	–	*r* = 0.870; *p* < 0.001	–
Corrected leg girth	–	*r* = 0.730; *p* = 0.003	–	*r* = 0.823; *p* < 0.001	–
BMI	–	*r* = 0.562; *p* = 0.037	–	*r* = 0.631; *p* = 0.016	–
∑8 Skinfolds	*r* = − 0.606; *p* = 0.022	–	*r* = − 0.554; *p* = 0.040	–	*r* = 0.695; *p* = 0.006
Fat mass (%)	*r* = − 0.631; *p* = 0.016	–	*r* = − 0.615; *p* = 0.019	–	*r* = 0.694; *p* = 0.006
Muscle mass (%)	–	–	*r* = 0.535; *p* = 0.048	–	–
Fat mass (Kg)	*r* = − 0.549; *p* = 0.042	–	–	–	*r* = 0.647; *p* = 0.012
Muscle mass (Kg)	–	*r* = 0.815; *p* = 0.001	–	*r* = 0.920; *p* = 0.001	–
Muscle-bone index	-	*r* = 0.598; *p* = 0.024	–	*r* = 0.603; *p* = 0.022	–

In the 13-years-old group (Table 9), long jump test showed positive moderate correlations with sitting height and bi-styloid breadth (*r* = 0.534–0.686; *p* = 0.005–0.040), and negative moderate correlations with ∑8 skinfolds and fat mass percentage (*r* =  − 0.427, −0.522; *p* = 0.002–0.046); medicine ball throw test showed positive moderate to high correlation with bone and muscle related variables (*r* = 0.566–0.702; *p* = 0.004 − 0.028); CMJ showed negative moderate correlations with fat-related variables and muscle-bone index (*r* =  − 0.544,−0.594; *p* = 0.020–0.036); CMJ power showed positive moderate to high correlations with all the analyzed variables (*r* = 0.521–0.855; *p* < 0.001 − 0.046), except with the ∑8 skinfolds, fat mass percentage and muscle to bone index; sprint test showed negative moderate correlations with muscle and fat related variables (*r* =  − 0.527, −0.625; *p* = 0.009–0.043); agility test showed negative moderate correlations with bone related variables (*r* =  − 0.300, −0.555; *p* = 0.032–0.038).

In the 14-years-old group (Table 10), medicine ball throw test showed a moderate positive correlation with sitting height (*r* = 0.699; *p* = 0.017; CMJ test showed a negative moderate correlation with the ∑8 skinfolds (*r* =  − 0.644; *p* = 0.024); CMJ power showed a positive moderate to high correlation with all the variables included (*r* = 0.603–0.901; *p* < 0.001–0.038); sprint and agility tests showed a positive moderate to high correlation with muscle, bone and fat related variables (*r* = 0.625–0.883; *p* < 0.001–0.030), except for the muscle mass correlation with agility, that was negative and high (*r* =  − 0.768; 0 = 0.004).

In the case of the 15-years-old group (Table 11), long jump test showed negative moderate correlations with ∑8 skinfolds, fat mass percentage and muscle mass (kg) (*r* =  − 0.549, −0.631; *p* = 0.016–0.042); both medicine ball throw and CMJ power showed positive moderate to high correlations with all the anthropometric variables (*r* = 0.545–0.920, *p* < 0.001–0.044), except with the ∑8 skinfolds and the fat and muscle mass percentages; CMJ test showed negative moderate correlations with the ∑8 skinfolds and fat mass percentage (−0.554, −0.615; *p* = 0.019–0.040), while a positive moderate correlation was observed with muscle mass percentage (*r* = 0.535; *p* = 0.048); the sprint test showed positive moderate correlations with the fat related variables (*r* = 0.647–0.695; *p* = 0.006–0.012).

The biological maturation showed positive moderate to high correlations with the physical fitness test in all the age groups (*r* = 0.604­-0.915; *p* < 0.001–0.017), except in the CMJ in the 12-years-old group, where the correlation was negative and high (*r* =  − 0.760; *p* = 0.047).

Tables 12 to 15 show the linear regression models in relation to the fitness tests, as well as the resulting predictive equations for each physical fitness test for each age group. Between one and three prediction models were found for the performance in the different fitness tests in the four age groups, which could explain 31 to 95% of the cases based on the anthropometric variables (*p* < 0.001–0.032). In the 12-years-old group, the most determinant variables in relation to performance were the maturity offset and body mass index (Table 12); in the 13-years-old group, the sitting height, corrected girths, maturity offset, bi-styloid breadth, body mass, muscle mass percentage, muscle-bone index, ∑8 skinfolds were the most determinant anthropometric variables (Table 13); in the 14-years-old group, the sitting height, ∑8 skinfolds, body mass, fat mass percentage and corrected arm girth were the most determinant anthropometric variables (Table 14); and in the 15-years-old group, fat mass percentage, corrected arm girth, sitting height, bone breaths, muscle mass percentage and ∑8 skinfolds were the most determinant anthropometric variables (Table 15).

**Table 12:** Regression models of the performance in the different physical fitness tests in the 12-years-old group.

Age group	Variable	Analysis	R^2^	*p* value	Included Independent Variables	SC	Equation
12 years old	CMJ	Model 1	0.58	0.047	Maturity offset	−0.76	CMJ = 6.479 − 21.229*maturity offset
	CMJ power	Model 1	0.87	0.002	BMI	0.93	CMJ power = 128.328 + 22.052*BMI

**Notes.**

SC Standardized coefficients

**Table 13:** Regression models of the performance in the different physical fitness tests in the 13-years-old group.

Age group	Variable	Analysis	R2	*p* value	Included independent variables	SC	Equation
13 years old	Long jump	Model 1	0.47	0.005	Sitting height	0.69	Long jump = −1.390 + 0.038*sitting height
				Model 2	0.74	<0.001	Sitting height	0.99	Long jump = − 0.550 + 0.055*sitting height − 0.075*corrected leg girth
							Corrected leg girth	−0.60	
				Model 3	0.82	<0.001	Sitting height	0.82	Long jump = −0.356 + 0.045*sitting height − 0.085*corrected leg girth + 0.190*bi-styloid breath
											Corrected leg girth	−0.69	
			Bi-styloid breath				0.38		
		Medicine ball throw	Model 1	0.63	<0.001	Maturity offset	0.79	Medicine ball throw = 5.332 + 1.544*maturity offset
		CMJ	Model 1	0.35	0.020	∑8 skinfolds	−0.59	CMJ = 33.668 − 0.076*Sum of 8 skinfolds
		CMJ power	Model 1	0.73	<0.001	Body mass	0.85	CMJ power = 124.236 + 9.072*body mass
				Model 2	0.83	<0.001	Body mass	1.48	CMJ power = 395.396 + 15.715*body mass − 30.141*corrected arm girth
							Corrected arm girth	−0.70	
				Model 3	0.89	<0.001	Body mass	2.00	CMJ power = −541.713 + 21.252*body mass − 42.547*corrected arm girth + 23.420*muscle mass (%)
											Corrected arm girth	−0.98	
			Muscle mass (%)				0.39		
		20 m sprint	Model 1	0.42	0.009	Muscle-bone index	0.65	Sprint = 2.806 + 0.561*muscle-bone index
	Agility test	Model 1	0.31	0.032	Bi-styloid breath	−0.55	Agility = 14.275 − 0.982*bi-styloid breadth

**Notes.**

SC Standardized coefficients

**Table 14:** Regression models of the performance in the different physical fitness tests in the 14-years-old group.

Age group	Variable	Analysis	R2	*p* value	Included independent variables	SC	Equation
14 years old	Medicine ball throw	Model 1	0.45	0.017	Sitting height	0.67	Medicine ball throw = −16.293 + 0.260*sitting height
		CMJ	Model 1	0.41	0.024	∑8 skinfolds	−0.64	CMJ = 35.526 − 0.421* ∑8 skinfolds
		CMJ power	Model 1	0.89	<0.001	Body mass	0.94	CMJ power =144.949 + 9.810*body mass (kg)
				Model 2	0.95	<0.001	Body mass	1.43	CMJ power = −59.752 + 14.930*body mass − 1.486* ∑8 skinfolds
			∑8 skinfolds				−0.55		
		20 m sprint	Model 1	0.78	<0.001	Fat mass (%)	0.88	Sprint =3.492 + 0.023*fat mass (%)
	Agility test	Model 1	0.61	0.003	Corrected arm girth	0.78	Agility =4.812 + 0.168*corrected arm girth

**Notes.**

SC Standardized coefficients

**Table 15:** Regression models of the performance in the different physical fitness tests in the 15-years-old group.

Age group	Variable	Analysis	R2	*p* value	Included independent variables	SC	Equation
15 years old	Long jump	Model 1	0.40	0.016	Fat mass (%)	−0.47	Long jump = 2.555 − 0.025*fat mass (%)
				Model 2	0.59	0.008	Fat mass (%)	−0.53	Long jump = 1.694 − 0.0.35*fat mass (%) + 0.042*corrected arm girth
			Corrected arm girth				0.49		
		Medicine ball throw	Model 1	0.83	<0.001	Sitting height	0.91	Medicine ball throw = −19.967 + 0.296*sitting height
		CMJ	Model 1	0.38	0.019	Fat mass (%)	−0.61	CMJ = 43.695 − 0.744*fat mass (%)
				Model 2	0.78	<0.001	Fat mass (%)	−1.04	CMJ = −73.071 − 1.259*fat mass (%) + 12.495*femur breadth
			Femur breadth				0.76		
		CMJ power	Model 1	0.87	<0.001	Biacromiale breadth	0.93	CMJ power = −1219.122 + 54.512*biacromiale breadth (kg)
				Model 2	0.93	<0.001	Biacromiale breadth	1.03	CMJ power = −2215.952 + 60.481*biacromiale breadth + 17.659*muscle mass (%)
			Muscle mass (%)				0.27		
		20 m sprint	Model 1	0.48	0.006	∑8 skinfolds	0.69	Sprint = 3.400 + 0.004* ∑8 skinfolds
				Model 2	0.65	0.003	∑8 skinfolds	1.02	Sprint = 4.401 + 0.006* ∑8 skinfolds − 0.058*biileocrestal breadth
	Biileocrestal breadth		−0.52						

**Notes.**

SC Standardized coefficients

## Discussion

One of the objectives of the present research was to analyse the differences between maturation groups in anthropometric variables in adolescent volleyball athletes. Significantly higher values were found in early maturers compared to average and late maturers in body mass, height, arm span and sitting height. These results are consistent with previous studies carried out in the adolescent male athlete population, which also found that subjects whose maturation process was more advanced showed higher values in these variables (Arede et al., 2019; López-Plaza et al., 2017b). The differences found between maturation stages in anthropometric variables could be related to the hormonal changes that take place around APHV (Malina & Bouchard, 1991). Previous studies have observed that both sex hormones and growth hormone (GH) increase dramatically in concentration during this stage (Handelsman, Hirschberg & Bermon, 2018; Malina & Bouchard, 1991). Sex hormones play an important role in the accumulation of adipose tissue and lean mass (Handelsman, Hirschberg & Bermon, 2018), which could explain the differences found in body mass. On the other hand, height and sitting height is markedly influenced by GH (Saenger, 2003), which could explain the higher values obtained by the subjects in the early maturers group. Similarly, the early maturers group obtained higher results in arm span and upper limb length. While in the early stages of growth, children experience cephalo-caudal and proximal-distal development (Malina & Bouchard, 1991), during adolescence growth occurs first in the limbs (Malina & Bouchard, 1991). This distal-proximal order in development could explain the differences shown between the maturation groups.

Also, significant differences were observed in adiposity-related variables (fat mass and percentage, and ∑6 and ∑8 skinfolds), between the early maturers and average maturers, and between early and late groups (fat mass), with higher values in the more mature subjects. These results are similar to those found in previous studies in which it was observed that in the adolescent athlete population, early maturers had a greater amount of adipose tissue (Albaladejo-Saura et al., 2021). The accumulation and distribution of adipose tissue undergoes changes during the adolescent stage in relation to sex hormones (Sandhu et al., 2005). In this case, a greater accumulation of adipose tissue seems to be related to an earlier onset of maturation in males (Sandhu et al., 2005), this could explain why, in this study, the early maturers showed a higher fat mass.

Regarding the other tissues of body composition, it was found that the group of early maturers showed significantly higher values than the average and late maturers in the components related to muscle development (muscle mass and corrected girths) and bone development (upper limb length and breadths). Previous research has shown that early maturers also have higher fat free mass and muscle mass than their peers (López-Plaza et al., 2017b). Muscle mass has been shown to be of great importance in sports performance (Fitts, McDonald & Schluter, 1991). The development of muscle mass appears to be linked to biological maturation, as the increase in muscle mass during adolescence is related to the increase in circulating testosterone, which in the male population can be up to 30 times higher than baseline values (Handelsman, Hirschberg & Bermon, 2018). Bone tissue has also been shown to be of great use in sports performance, serving as a structure for muscle development (Holway & Garavaglia, 2009). Bone development occurs with a marked increase in the pubertal stage, influenced by GH, and then increases gradually into adulthood (Ohlsson et al., 1998). This increase in the hormones responsible for the increase in bone and muscle mass around APHV could be the explanation for the differences found between maturation groups in this study.

When age was introduced as a covariable, a significant influence was observed in the differences found between all groups in the basic measurements, bone variables and muscle variables, while in the fat variables only influence was observed in the differences between early and average maturers, with the early maturers group showing higher values. This phenomenon has been described in previous works analyzing the effect of age in the anthropometric variables. Valente-Dos-Santos et al. (2014) observed that height and fat free mass increased with age in all the maturation groups, with higher values in the more mature individuals, while the fat mass differences were smaller. In the present paper, the analyzed influence of age on the significant differences may be related to the fact that the sample of the present study had an age close to the APHV, which typically occurs in boys around 13.8 ± 1.0 years of age (Malina & Bouchard, 1991; Rommers et al., 2019; Sherar et al., 2005), as it is in this period that the most notable changes produced by growth spurt occur, with an increase in muscle and bone tissue as age progresses (Handelsman, Hirschberg & Bermon, 2018; Malina & Bouchard, 1991).

Taking into account that height, arm span and leg length are important factors in volleyball performance (Zhao et al., 2019), it could be hypothesized based on the results obtained that early maturers might have a competitive advantage in adolescent stages that could be neutralised by average and late maturers when they reach adult size. In that case, this is an issue that would need to be considered in volleyball talent identification models.

Another of the objectives of this article was to compare the maturation groups in terms of performance in the physical fitness tests. The selection of the tests included in the present study was made taking into account the most determinant physical abilities in young male volleyball players, such as the power of upper and lower limbs (Tsoukos et al., 2019), based on previous studies that have used the tests in a similar population of adolescent volleyball players (Albaladejo-Saura et al., 2022) and in other sports (Arede et al., 2019; Castro-Piñeiro et al., 2013; Katić, Grgantov & Jurko, 2006; López-Plaza et al., 2017b). It was shown that the early maturers performed better in the medicine ball throw and CMJ power than their peers in the average and late maturers groups, and that the average maturers performed better than the late maturers. Among the factors that positively affect the production of muscle power, it has been observed that one of the key factors is muscle mass, with a relationship existing between the increase of muscle mass and the production of power (Fitts, McDonald & Schluter, 1991). Since both tests are related to the amount of muscle mass, and in addition, the CMJ power is also related to body mass, the significant differences found in these anthropometric variables between maturation groups may help to understand why these differences were found in the physical fitness tests. On the other hand, the flexibility tests showed no significant differences between groups. This could be caused by flexibility is not a physical capacity highly influenced by the maturational process (Albaladejo-Saura et al., 2021), and yet it could be more influenced by the adaptations produced by volleyball training, as extensibility seems to be sensitive to the changes produced by training, improving it and producing morphological and neurological adaptations (Klaver et al., 2018), without the influence of the maturity status on this adaptations (Albaladejo-Saura et al., 2021). The results of the present research are in line with those found in male adolescents, in which it has been found that the early maturers groups have better results than the average and late maturers in muscle strength and power-dependent tests, but there were no differences in the flexibility tests between maturation groups (Albaladejo-Saura et al., 2021). In this sense, Arede et al. (2019), observed that, in a sample of basketball players of a similar age to the one included in our study, the more mature players performed better in the medicine ball throw and CMJ power, but not in CMJ jump height. Similarly, López-Plaza et al. (2017a), observed that more mature players in a sample of kayakers performed better in the aforementioned tests than their chronological age peers.

Inclusion of the covariate age showed a significant influence on differences in medicine ball throw, CMJ power, 20 m sprint and agility tests performance. However, the pairwise analysis of the differences only showed statistical significance in the medicine ball throw and CMJ power, obtaining better results the players whose maturation process was more advanced. The influence of age has been demonstrated in previous studies, in which it has been observed that as age advances, performance in physical fitness tests improves (Rommers et al., 2019; Valente-Dos-Santos et al., 2014), especially as young athletes approach to the APHV due to the physiological and morphological changes that occur around these stages (Handelsman, Hirschberg & Bermon, 2018; Malina & Bouchard, 1991).

As a result of the present research, sports talent identification programmes could include assessments of these capacities, having to relativize exclusively the results of upper limbs and jumping power according to the adolescent’s maturational state, as maturation does not seem to affect the other factors in isolation but rather in combination with age. However, it may be considered that these tests are not strictly volleyball specific, so further research in this topic including volleyball related performance test would be of great interest.

Another objective of the present research was to determine which of the variables analyzed could best predict performance in the physical fitness tests. It was found that fat mass percentage predicted worse performance in the long jump, sprint and agility tests. This may be due to the fact that in physical capacities characterized by explosive movements, added weight in the form of adipose tissue can weigh down performance by requiring greater effort for the displacements (Albaladejo-Saura et al., 2021). However, absolute adipose tissue related variables, such as fat mass in kilograms or skinfold sums, did not showed contribution to performance, nor positive or negative in the results showed in the regression analysis in the aforementioned tests. This may be because, as observed in previous research, physical exercise can modify fat mass percentages without significant differences in absolute fat mass as a consequence of the increase of muscle mass (Cruz-Ferreira, Lino & Azevedo, 2009; Vaquero-Cristóbal et al., 2016). Similar results have been found in previous researches, finding that adipose tissue in absolute amounts could not be the most determinant variable that contributes to sport performance related to long-jump in adolescent population, as it has been observed that those subjects who were taller and had longer upper and lower limbs performed better than their peers (Hraski et al., 2015). Even though more mature players showed a higher amount of fat mass and higher values of adipose tissue related variables than their peers, it seems that relative values of fat mass, represented as percentage, are more relevant for the sport performance in the selected tests in young male volleyball players.

On the other hand, age, maturity offset and structural variables, such as height, sitting height and iliospinal height; and variables related to muscle development, such as muscle mass (kg) and corrected arm and calf circumferences, are predictors of better performance in the long jump, medicine ball throw, CMJ power and agility tests. Previous studies have already pointed out the importance of bone structure in physical performance, due to its relationship with the biomechanical parameters of strength execution and for providing the appropriate environment for better muscle development (Holway & Garavaglia, 2009). The muscle mass is a key factor to improve the performance in the physical abilities where body mass shifts in the horizontal or vertical plane, such as those that determine the volleyball performance (Sarro et al., 2019).

When the regression analysis was performed dividing the sample by age groups, the same direction of the results was observed, showing the bone and muscle related variables to be predictors of better performance in those physical fitness variables related to the strength and power production in all the age groups, and fat related variables being related with worse performance. The body mass was a predictor of better power production in the CMJ test, result that is in line with previous research, as the power calculation depends on the athletes body mass (Jeras, Bovend’Eerdt & McCrum, 2020). However, CMJ height, sprinting and agility were negatively related to subcutaneous fat and related variables in the 13-, 14- and 15-year-old group, which is in line with previous research (Albaladejo-Saura et al., 2021; Chena Sinovas et al., 2015). It is noteworthy that maturity offset was one of the predictor variables for performance in the 12- and 13-years-old groups, in relation to the CMJ and medicine ball throw tests. These age groups are, in terms of chronological age, before the theoretical time of the APHV, which in boys has been documented at around 14 years of age, with individual variations (Malina & Bouchard, 1991). Previous research has described that the differences found in physical performance in adolescent athletes tend to equalize as chronological age advances, especially from the age of 14 years, after all the subjects had passed the APHV, until adulthood (Dugdale, McRobert & Unnithan, 2021; Dugdale et al., 2021). This could be the reason why maturity offset is a predictor of performance at these ages, but was not a predictor of performance in the 14- and 15-years-old groups. It may be noticed that some of the variables that showed significant correlations when the whole group was analyzed did not seem to have correlations in the age groups, perhaps due to the small sample included in each one. The correlations and the influence of anthropometric variables in the performance in fitness test in adolescent athletes should be investigated deeply with bigger samples of different ages. Further research is also needed to clarify if these variables related to bone and muscle mass allow the differentiation of the players according to their sport level once the adult development is reached.

However, the present research has some limitations. Firstly, the method used to establish the maturity status of the players was not the wrist and hand X-ray, considered the gold standard (Malina & Bouchard, 1991). However, despite being the gold standard, there are some considerations to be taken into account, as the X-ray methods are not without its problems. It has been proved that they expose the participants to a significant amount of radiation; are invasive, costly, and time intensive (Towlson et al., 2021). As a consequence of the potential problems of using this method, some authors have proposed using alternative less invasive methods in the adolescent population (Towlson et al., 2021).

Alternative methods have been developed to observe APHV based on repeated measures over time and the development of mathematical models, such as SITAR, which allow observation rather than estimation of peak growth during adolescence (Cole, 2018). However, this model can only be applied from longitudinal designs, so this was not an option for the present research due to its cross-sectional nature.

The other option is to use maturation estimating equations based on regression equations, which is a non-invasive method that is easy to apply in field research and has been widely used in recent sport science research (Albaladejo-Saura et al., 2021). However, equations may introduce some error in the calculation of the maturity offset, established at around 0.50–0.59, according with previous research, limiting its use to some extent (Malina et al., 2016; Malina et al., 2021; Mirwald et al., 2002). In fact, it has been noted in research that the estimating equations for maturity offset tend to underestimate the value for early maturers, while overestimating it for late maturers (Towlson et al., 2021). Among these, perhaps the most popularly used has been that of Mirwald et al. (2002), as in a recent systematic review with meta-analysis, out of seven studies that selected to assess somatic maturation through anthropometric equations, six were using Mirwald et al. (2002) equation to classify the athletes of different sports (Albaladejo-Saura et al., 2021). Although this equation shows the problems outlined above, some recommendations from previous studies to minimize its effect were followed. Specifically, although the original formula of Mirwald et al. (2002) was validated in a population with a wide age range (8–18 years old), and it has been observed that the results change more or less steadily with advancing chronological age (Malina et al., 2021). For this reason, some authors have recommended narrowing the age range of participants to 12–16 years old, as well as controlling for the effect of chronological age on the estimations (Malina et al., 2021; Towlson et al., 2017; Towlson et al., 2021). Furthermore, having demonstrated the potential issues of using equations based on anthropometric measures, it has been noted that they may have some utility when used to categorize participants as pre-, circum- or after-APHV (Malina et al., 2021; Mirwald et al., 2002; Towlson et al., 2021). Knowing the limitations of this method, this research uses the estimation of maturity offset as a categorical variable, includes participants with chronological age ranges within the recommended range, and uses the chronological age of the participants to control for the effect of differences between groups.

Other limitations of the present study are the cross-sectional research design, this prevented the use of APHV observation models as well as the establishment of a deeper relationship between anthropometric variables and physical fitness throughout adolescence and the sample size, which limits the extrapolation of the results to populations that do not have similar characteristics to the one included in the present study. Future research could address these limitations in longitudinal research designs, with larger samples, studying the influence of biological maturation on anthropometric and physical fitness variables. It should be recommendable also the use of different methods to approach the estimation or observation of the maturity status, that allow the researcher to include subjects of a wider age range in order to clarify the relationships between maturation and performance during the different stages.

## Conclusions

Early maturers showed higher values in measures such as height, body mass, arm span, sitting height, bone diameters, muscle perimeters and fat, muscle and bone masses, as well as in distance achieved in the medicine ball throw and in CMJ power. These differences found in favour of players whose maturation process was more advanced could represent an advantage in volleyball sport performance during adolescence with respect to their chronological age peers. When assessing anthropometric variables and the physical condition of young players, biological maturation should be considered as early maturers may have a competitive advantage. Similarly, attention should be paid to variables such as height, sitting height, iliospinale height and muscle girths, as they have been shown to have a high predictive power for performance in physical fitness tests related to volleyball requirements. It should also be taken into account that when players’ age is close to APHV, the differences found between maturation groups both in anthropometric and physical fitness performance are influenced by age. However, due to the small sample included and the limitations identified in the present research, these findings should be taken with caution, as they may only be applicable to the target population.

## Supplemental Information

10.7717/peerj.13216/supp-1 Supplemental Information 1 Database of the participants

## Additional Information and Declarations

### Data Availability

The following information was supplied regarding data availability: The raw measurements are available in the Supplementary File.
